# Polyamidoamine Dendrimers Functionalized Water-Stable Metal–Organic Frameworks for Sensitive Fluorescent Detection of Heavy Metal Ions in Aqueous Solution

**DOI:** 10.3390/polym15163444

**Published:** 2023-08-18

**Authors:** Dandan Guo, Nadeem Muhammad, Shuxin Yu, Jinhui Wang, Shaohua Huang, Yan Zhu

**Affiliations:** 1 Institute of Drug Discovery and Technology, Ningbo University, Ningbo 315211, China; 2 Department of Environmental Engineering, Wuchang University of Technology, Wuhan 430223, China; 3 Qian Xuesen Collaborative Research Center of Astrochemistry and Space Life Sciences, Ningbo University, Ningbo 315211, China; 4 Department of Chemistry, Xixi Campus, Zhejiang University, Hangzhou 310028, China

**Keywords:** metal–organic frameworks, polyamidoamine dendrimers, fluorescence detection, heavy metal ions

## Abstract

In this work, polyamidoamine (PAMAM)-functionalized water-stable Al-based metal–organic frameworks (MIL-53(Al)-NH_2_) were proposed with enhanced fluorescence intensity, and used for the sensitive detection of heavy metal ions in aqueous solution. The size and morphology of MIL-53(Al)-NH_2_ were effectively optimized by regulating the component of the reaction solvents. PAMAM dendrimers were subsequently grafted onto the surface with glutaraldehyde as a cross-linking agent. It was found that the size and morphology of MIL-53(Al)-NH_2_ have great influence on their fluorescence properties, and PAMAM grafting could distinctly further improve their fluorescence intensity. With higher fluorescence intensity, the PAMAM-grafted MIL-53(Al)-NH_2_ showed good linearity (R^2^ = 0.9925–0.9990) and satisfactory sensitivity (LOD = 1.1–8.6 μmol) in heavy metal ions determination. Fluorescence enhancement and heavy metal ions detection mechanisms were discussed following the experimental results. Furthermore, analogous water-stable Materials of Institute Lavoisier (MIL) metal–organic frameworks such as MIL-53(Fe)-NH_2_ were also proved to have similar fluorescence enhancement performance after PAMAM modification, which demonstrates the universality of the method and the great application prospects in the design of PAMAM-functionalized high-sensitivity fluorescence sensors.

## 1. Introduction

With the rapid development of industrialization, heavy metal contamination has prompted great concern all over the world. Compared to organic pollution, toxic heavy metal ions cannot be biodegraded and thus accumulate in surface water, soil environment and food. Over the past few decades, humans have been negatively affected by heavy metal ions due to their continuous enrichment [1]. Therefore, trace detection of heavy metal ions is of great significance.

Up to now, various technologies have been validated for the sensitive detection of heavy metal ions, including inductively coupled plasma mass spectrometry (ICP-MS), inductively coupled plasma optical emission spectroscopy (ICP-OES), inductively coupled plasma atomic emission spectroscopy (ICP-AES) and atomic absorption spectroscopy (AAS) [2,3,4,5]. However, these analytical technologies have many limitations such as being time consuming, complex procedures, requiring bulky instrumentation and professional expertise, which fail to meet the requirements of rapid and on-line detection [6,7]. Whereas, with the distinct merits of high sensitivity, economy, simplicity and real-time monitoring, the fluorescent method is considered to be an effective method for the rapid determination of heavy metal ions [8,9]. The design and preparation of fluorescent sensors is the key to fluorescence detection.

Metal–organic frameworks (MOFs) are a kind of hybrid material self-assembled by metal ion centers or clusters and organic ligands. They have a large specific surface area, adjustable pores, predictable nanostructure, high load capacity, good thermal and chemical stability, and show a wide range of applications in energy, separation, gas storage, drug delivery and sensing [10,11,12]. Over the past 10 years, many luminescent MOFs (LMOFs) have been proposed for the recognition and detection of heavy metal ions in solution. For these LMOFs, fluorescence originated from the mental center or organic ligands, and was influenced by both morphology and post-modifications [7,13,14]. In the detection process, fluorescent changes result from photoinduced electron transfer (PET), fluorescence resonance energy transfer (FRET), deprotonation or hydrogen-bond interaction between target metal ions and MOFs sensors [15,16,17]. Based on these mechanisms, Saedi et al. proposed fluorescent TMU-16 for detection of Cu^2+^ in aqueous solution [18]. Hou et al. proposed a reliable sensing platform for arsenic via direct post-synthetic modification of UiO-66 [14]. However, with the further development of metal ion detection technology, higher requirements are put forward for the stability and sensitivity of LMOFs sensors [19,20]. According to Kumar et al., Materials of Institute Lavoisier (MIL) is a typical MOFs material with high water stability [21], which is a good candidate for heavy metal ions detection in aqueous solution, whereas the detection sensitivity of these materials is limited in the previous work [22]. We think that surface functionalization can further improve the detection sensitivity of MIL MOFs materials.

Poly (amidoamine) (PAMAM) dendrimers are a kind of widely used macromolecular material with unique structural characteristics, including a regular three-dimensional structure, controllable molecule chains, extensive internal cavity and abundant functional terminal groups. Due to their special structure and properties, PAMAM dendrimers have been applied in various fields [23,24,25]. It is worth mentioning that the whole generation of PAMAM dendrimers are good candidates for post-functionalization of LMOFs [26]. With a large number of terminal amino groups and flexible aliphatic bonds, PAMAM-functionalized LMOFs are expected to have higher fluorescence intensity [27]. In addition, considering the good affinity between PAMAM and heavy metal ions [28], PAMAM-functionalized MOFs will have great potential in the high-sensitivity fluorescence detection of heavy metal ions.

Herein, aluminum-based MIL MOFs (MIL-53(Al)-NH_2_) were constructed with 2-aminoterephtalic acid as organic ligands via a solvent thermal method. The size, morphology and luminescence property of MIL-53(Al)-NH_2_ were optimized by varying the composition of the reaction solvent. Afterward, the optimized particles were modified with PAMAM dendrimers, which achieved obvious enhancement in fluorescence intensity. The obtained PAMAM-functionalized MOFs exhibited satisfactory performance for heavy metal ions determination. Based on these results, possible mechanisms for fluorescence enhancement and fluorescence detection were discussed in detail. Finally, the universality of this fluorescence enhancement approach was verified by the comparison of analogous MIL MOFs.

## 2. Materials and Methods

### 2.1. Chemical Reagents

N,N-dimethylformamide (DMF, 99.5%) and 2-aminoterephthalic acid (NH_2_-BDC, 98%) were purchased from Macklin Biochemical Co., Ltd. (Shanghai, China). Glutaraldehyde (AR, 50% in H_2_O), methyl acrylate (MA, 98.5), methanol (AR) and all metal salts of AR grade were purchased from Aladdin Chemical Co., Ltd. (Shanghai, China). Ethylenediamine was purchased from China National Medicines Corporation Ltd. (Beijing, China). All the solutions of mental ions were prepared by dispersing their corresponding chloride salt in deionized water.

### 2.2. Synthesis of Materials

#### 2.2.1. Synthesis of PAMAM Dendrimers in Different Generations

PAMAM dendrimers in different generations were synthesized referring to the previous literature reported by Tomalia et al. [29]. Firstly, 10 mL ethylenediamine and 55.6 g methyl acrylate were dissolved in 100 mL methanol solvent, and the Michael addition reaction was carried out at room temperature for 24 h. A half-generation PAMAM dendrimer (0.5G PAMAM) was obtained by subsequent rotary evaporation purification under the condition of 300 mbar and 60 °C. Subsequently, 54.0 g 0.5G PAMAM and 150 mL methanol solvent were placed into a flask, excess ethylenediamine was added and stirred overnight to obtain 1.0G PAMAM. Obviously, PAMAM dendrimers in different generations could be obtained by repeating the steps above. (Element analysis results are shown in Table S1.)

#### 2.2.2. Synthesis of MIL MOFs

The MIL MOFs materials were prepared by solvent thermal method, which links the organic metal center with the NH_2_-BDC regent [30]. Take MIL-53(Al)-NH_2_ for example: 0.76 g AlCl_3_·6H_2_O and 0.56 g NH_2_-BDC were dissolved in 30 mL solvents. The mixture was placed in a 50 mL Teflon-lined autoclave and kept at 150 °C for 24 h. The resulting products were separated from the reaction mixture by centrifugation and washed by DMF and methanol successively. Finally, the obtained products were dried at 70 ºC in vacuum overnight. To obtain MIL-53(Al)-NH_2_ in various morphologies, deionized water was added into DMF solvents in the proportion of 0%, 25%, 50% and 75%, respectively. Other MIL MOFs (MIL-53(Fe)-NH_2_) were synthesized by the same method [31], which is specified in the Supplementary Materials.

#### 2.2.3. Preparation of PAMAM-Grafted MOFs Materials

**Figure 1:**
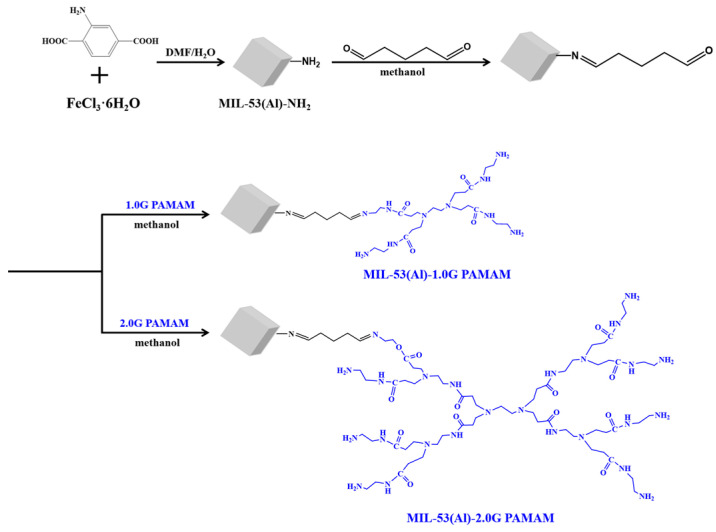
Preparation of PAMAM-grafted MOFs materials.

As for the preparation of PAMAM-grafted MOFs materials, 300 mg MIL MOFs were dispersed in 100 mL methanol solvent containing 5% glutaraldehyde, and the reaction was carried out at room temperature for 3 h. The resulting glutaraldehyde-grafted MOFs were washed three times with the reaction solvent. Afterward, the obtained products were treated with 1 mL PAMAM dendrimers in 30 mL methanol solvent. After carrying out the reaction for 2 h at room temperature, the resulting PAMAM-grafted MOFs materials were fully washed with methanol and dried in vacuum overnight. To explore the effect of PAMAM generations on the fluorescence properties of MOFs materials, 1.0G PAMAM and 2.0G PAMAM were utilized for the modification of MIL-53(Al)-NH_2_ and named MIL-53(Al)-1.0G PAMAM and MIL-53(Al)-2.0G PAMAM, respectively (Figure 1).

### 2.3. Characterization

The sizes and morphologies of the MOFs materials were studied using scanning electron microscopy (Zeiss Sigma 300, Oberkochen, Germany). The X-ray diffraction (XRD) patterns were obtained by a diffractometer (Ultima IV, Tokyo, Japan). Fourier transform infrared (FTIR) spectra were collected on an IR spectrophotometer (ThermoFisher iN 10, Waltham, MA, USA). The valence states of contained elements and surface metal ions of the MOFs were affirmed with an X-ray photoelectron spectroscopy (XPS) instrument (Thermo Scientific K-Alpha, Waltham, MA, USA). EDS-mapping analysis of the quenched MOFs was evaluated by a transmission electron microscope (EOL JEM 2100F, Tokyo, Japan). All fluorescence tests were performed on a fluorescence spectrophotometer (Hitachi F-7100, Tokyo, Japan).

### 2.4. Luminescent Measurements

In order to investigate the influence of morphology, PAMAM dendrimers, pH and concentrations on the fluorescence properties of the PAMAM-grafted MOFs materials, fluorescent determination experiments were conducted at room temperature with 370 nm as the excitation wavelength. The experiment was carried out by controlling a single variable method, concentrations of the synthesized materials varied from 0.01 mg mL^−1^ to 1 mg mL^−1^, and the pH of the solutions was adjusted from 4 to 10.

To evaluate the sensitivity and selectivity of the PAMAM-grafted MOFs materials to metal ions, selected ions (Pb^2+^, Cu^2+^, Fe^3+^, Co^2+^, Zn^2+^, K^+^, Na^+^, Mg^2+^) were added to 1 mg mL^−1^ MIL-53(Al)-2.0G PAMAM solutions. The pH of solutions was adjusted to 7 by 0.1 mM HCl and 0.1 mM NaOH. After incubation for 5 min, fluorescence intensities for the mixture were measured under the excitation wavelength of 370 nm.

## 3. Results and Discussion

### 3.1. Morphology and Structure of MIL-53(Al)-NH_2_

**Figure 2:**
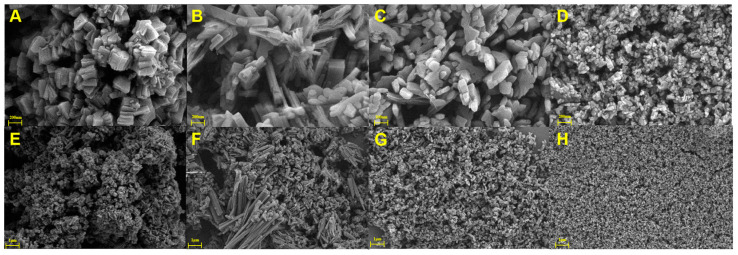
SEM images of MIL-53(Al)-NH_2_ obtained with a water proportion of 0% (**A**,**E**), 25% (**B**,**F**), 50% (**C**,**G**) and 75% (**D**,**H**).

It is well known that the composition of a solvent has a big impact on the solvothermal reaction. In this work, four different MIL-53(Al)-NH_2_ materials were prepared in mixed solvents with different DMF and deionized water ratios. The morphologies of the obtained products were investigated by scanning electron microscopy (SEM). According to the results in Figure 2, MIL-53(Al)-NH_2_ prepared without deionized water (0% H_2_O) consisted of aggregated granular particles (Figure 2A,E). With the increasing proportion of water, particle size decreased gradually and the morphology became irregular (Figure 2B–D,F–H), indicating that the water proportion has a great impact on the morphology and particle size of the MIL-53(Al)-NH_2_. These results could be interpreted as different metal coordination capacities between DMF and the water solvent [32].

**Figure 3:**
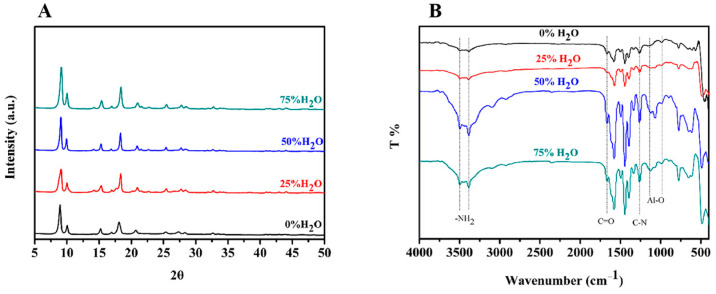
(**A**) XRD patterns of as-synthesized MIL-53(Al)-NH_2_ with different proportions of water; (**B**) FTIR spectra of MIL-53(Al)-NH_2_ materials synthesized with different proportions of water.

In addition to morphology characteristics, the crystal structure of MIL-53(Al)-NH_2_ products were characterized by powder X-ray diffraction (PXRD) analysis. The XRD patterns of the materials obtained in different solvents are shown in Figure 3A. The results showed that the crystal structures of the MIL-53(Al)-NH_2_ products were identical despite the different reaction solvents. Therefore, we can assume that the solvent composition has little impact on the crystal structure of the products. Furthermore, the XRD spectra were in good agreement with previous research [33], which implies that the MIL-53(Al)-NH_2_ MOFs were successfully synthesized.

Finally, FITR spectra of the MIL-53(Al)-NH_2_ materials were collected by an FTIR spectroscope from 4000–400 cm^−1^ (Figure 3B). As the results show in Figure 3B, two obvious peaks at 3482 and 3376 cm^−1^ corresponded to symmetric and asymmetric stretching vibrations of the N-H bond, evidence that the -NH_2_ had been well introduced after the coordination reaction between BDC-NH_2_ and Al^3+^, which contributed to the solubility and reactivity of MIL-53(Al)-NH_2_. The adsorption band at 1669 cm^−1^ resulted from the vibration of C=O and the band at 1260 cm^−1^ was attributed to the adsorption of the C-N bond, indicating that NH_2_-BDC was linked into the framework of the NH_2_-MIL-53(Al) nanoplates. The wide peak at 1120–1000 cm^−1^ was related to Al-O, and further confirmed that the O atoms within NH_2_-BDC had been linked to Al^3+^, and the MIL-53(Al)-NH_2_ framework had been established [7]. In addition, the spectra of the MOFs materials synthesized in four different solvents were identical, from which it could be concluded that the reaction solvent has little effect on the composition of a MOFs material.

According to the above characterization results, the composition of the reaction solvent has a great influence on the size and morphology of MIL-53(Al)-NH_2_ materials, but does not affect their crystal structure and surface functional groups.

### 3.2. Characterization of PAMAM-Grafted MOFs Materials

**Figure 4:**
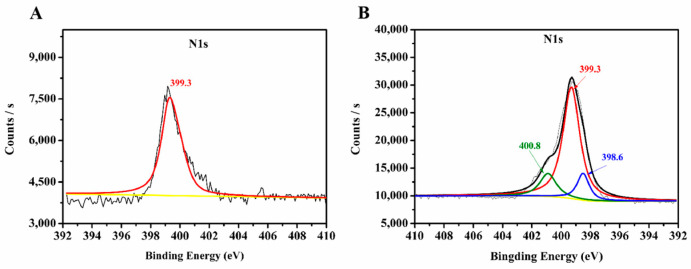
XPS spectra of nitrogen in (**A**) MIL-53(Al)-NH_2_ and (**B**) PAMAM-grafted MIL-53(Al)-NH_2_. The yellow line and black lines in the figure are the baseline and the total result of all peak fits, respectively.

In this work, PAMAM dendrimers were utilized for the surface modification of the prepared MIL-53(Al)-NH_2_ materials. Functionalization with PAMAM dendrimers greatly changed the surface groups of MIL-53(Al)-NH_2_ materials, which was directly reflected in the XPS analysis. N1s spectra of MIL-53(Al)-NH_2_ and MIL-53(Al)-2.0G PAMAM are shown in Figure 4. The observed 399.3 eV peak in raw MIL-53(Al)-NH_2_ materials (Figure 4A) was attributed to free amine groups [34]. After grafting with PAMAM dendrimers, two additional peaks at a higher binding energy (400.8 eV) and lower energy (398.6 eV) were observed in MIL-53(Al)-2.0G PAMAM (Figure 4B), which could be assigned to amide bond and Schiff base structure [35,36] in PAMAM, respectively. The obtained results indicated that PAMAM dendrimers have been grafted onto MIL-53(Al)-NH_2_ materials successfully.

### 3.3. Fluorescence Properties

The fluorescence properties of raw MIL-53(Al)-NH_2_ in different morphologies and of MIL-53(Al)-NH_2_ grafted with PAMAM in different generations (MIL-53(Al)-1.0G PAMAM and MIL-53(Al)-2.0G PAMAM) were determined. All of the materials have an obvious fluorescence emission around 440 nm under excitation at 370 nm. In order to obtain the best fluorescence detection conditions, the influences of pH and concentration were also investigated.

#### 3.3.1. Influence of Morphology

**Figure 5:**
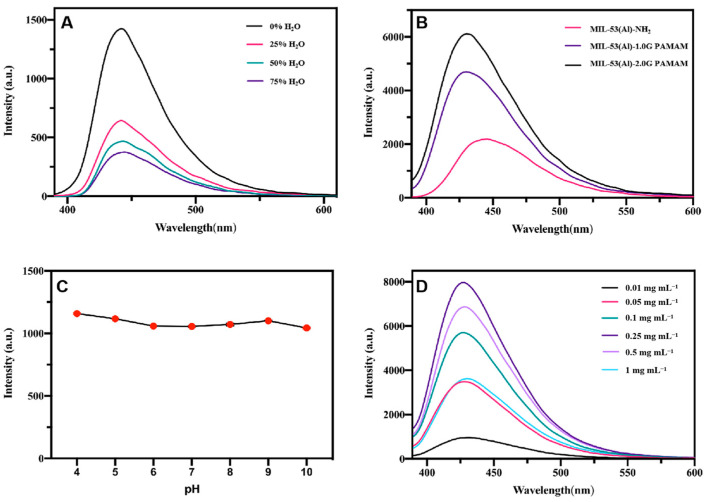
(**A**) Fluorescence emission spectra of MIL-53(Al)-NH_2_ synthesized with different proportions of water (excitation slit: 2.5 nm; emission slit: 5.0 nm); (**B**) Fluorescence emission spectra of MIL-53(Al)-NH_2_ grafted with PAMAM dendrimers (excitation slit: 2.5 nm; emission slit: 2.5 nm); Effect of pH (**C**) and concentrations (**D**) on fluorescence intensity of MIL-53-1.0G PAMAM (excitation slit: 1.0 nm; emission slit: 10 nm).

According to the above discussion, MIL-53(Al)-NH_2_ in different morphologies were synthesized, due to the different metal coordination capacities between DMF and the water solvent. To investigate the effect of morphology on fluorescence properties, emission spectra of the MIL-53(Al)-NH_2_ prepared in different solvents (0% H_2_O, 25% H_2_O, 50% H_2_O, 75% H_2_O) were characterized under the same condition. As the results show in Figure 5A, the fluorescence intensity of different materials exhibited the same trend with the increase of excitation wavelength, but the intensity was different. The similar emission spectrum could be considered as part of the same mechanism that resulted in electric transitions relating to the surface states. Different fluorescence intensities suggested that the fluorescence intensities of MIL-53(Al)-NH_2_ MOFs were affected by their reaction solvents in the synthesis process, probably due to the difference in size and morphology of the obtained products [37]. Because of the maximum fluorescence intensity, the MIL-53(Al)-NH_2_ synthesized in pure DMF (0% H_2_O) was selected for further modification.

#### 3.3.2. Influence of PAMAM Dendrimers

To investigate the effect of PAMAM dendrimers, the fluorescence properties of MIL-53(Al)-NH_2_, MIL-53(Al)-1.0G PAMAM and MIL-53(Al)-2.0G PAMAM were determined at the same time, and the result is shown in Figure 5B. Compared to MIL-53(Al)-NH_2_, the fluorescence intensity of MIL-53(Al)-1.0G PAMAM and MIL-53(Al)-2.0G PAMAM increased dramatically, accompanied by a blue shift. Meanwhile, the fluorescence enhancement increased with higher PAMAM generations. Generally, PAMAM grafting transformed the surface amino groups of MIL-53(Al)-NH_2_ into Schiff base, which should have resulted in the red shift of emission spectrum. However, more terminal amino groups in PAMAM dendrimers caused blue shift at the same time. Considering the comprehensive influence of the two factors, the emission peak of PAMAM-grafted MIL-53(Al)-NH_2_ exhibited slight blue shift. As for the enhancement of fluorescence intensity by PAMAM, high-branched structure and large electron clouds of PAMAM dendrimers could lead to efficient intramolecular charge transfer (ICT) and thus enhance the fluorescence intensity of MOFs materials [38,39].

#### 3.3.3. Influence of pH and Concentration

As fluorescence properties of LMOFs would be affected by pH and concentration, fluorescence intensities of PAMAM-grafted MIL-53(Al)-NH_2_ were tested in aqueous solutions with different pHs (pH = 4–10) and concentrations (0.01–1 mg L^−1^). As indicated in Figure 5C, the fluorescence intensity of MIL-53(Al)-1.0G PAMAM materials were relatively pH-independent in the range of 4 to 10. This property being identical to raw MIL-53(Al)-NH_2_ materials implied that PAMAM grafting did not change the acid-base stability of MIL-53(Al)-NH_2_ [40]. However, according to the results in Figure 5D, the fluorescence intensity gradually increased with increasing concentration of MIL-53(Al)-1.0G PAMAM solutions. When the concentration was 0.25 mg mL^−1^, the fluorescence intensity reached a maximum. Subsequently, the fluorescence intensity decreased with increasing concentration, which could be interpreted as self-quenching behavior [41].

### 3.4. Sensing of Metal Ions

#### 3.4.1. Possible Application for Metal Ions Detection

On the basis of their fluorescence properties, PAMAM-grafted MIL-53(Al)-NH_2_ were applied for metal ions detection. Various kinds of metal ions (1 mmol L^−1^) were added to a certain concentration of MIL-53(Al)-2.0G PAMAM solution (pH = 7). The fluorescence quenching efficiency (QE) was calculated by the following equation:

QE = (F_0_ − F)/F_0_ (1)

where F_0_ and F are the fluorescence intensities of the MOFs solutions before and after metal ion quenching, respectively. As displayed in Figure S1, common metal ions have little response to MIL-53(Al)-2.0G PAMAM, while heavy metal ions exhibited different effects on the fluorescence intensity. In particular, Pb^2+^, Cu^2+^, Fe^3+^ and Co^2+^ quenched the fluorescence dramatically. Such results implied that MIL-53(Al)-2.0G PAMAM materials have great potential for high-sensitivity detection of heavy metal ions in aqueous solution.

#### 3.4.2. Detection of Heavy Metal Ions

**Figure 6:**
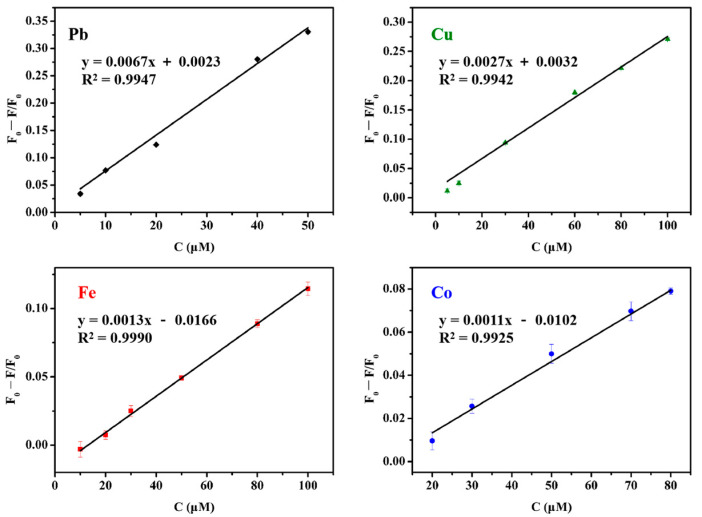
Linear relationships between heavy metal ions concentrations and fluorescence quenching efficiency.

The fluorescence responses of MIL-53(Al)-2.0G PAMAM toward Pb^2+^, Cu^2+^, Fe^3+^ and Co^2+^ were investigated at different concentrations. Relationships between metal ions concentrations and fluorescence quenching efficiency were shown in Figure 6 and Figure S2. The limit of detection (LOD) was calculated according to three times the F_0_ standard deviation [7]. Good linear relationships were obtained with linear correlation coefficients (R^2^) ranging from 0.9925 to 0.9990. The LOD of Pb^2+^, Cu^2+^, Fe^3+^ and Co^2+^ were 1.1 μmol, 1.1 μmol, 2.9 μmol and 8.6 μmol, respectively. Comparing to the reported MIL LMOFs, PAMAM grafting improved the detection sensitivity effectively (Table 1).

To evaluate the practicability of MIL-53(Al)-2.0G PAMAM fluorescence sensors, 0.05 mM Pb^2+^ and Fe^3+^ were added to tap water, drinking water and tea samples and detected. Recovery rates of the added ions are shown in Table 2. Satisfactory results were obtained in tap water (88.2–95.4%) and drinking water (94.0–95.0%). However, the recovery rates in tea samples were lower than that in tap water and drinking water; the complex ingredients in tea samples always display strong background UV absorption and fluorescence, and weaken the response signal of MIL-53(Al)-2.0G PAMAM [42]. Therefore, the proposed MIL-53(Al)-2.0G PAMAM fluorescence sensor has great feasibility for the determination of heavy metal ions. But the selectivity needs to be improved further.

#### 3.4.3. Sensing Mechanism

In the detection of heavy metal ions, the fluorescent responses of LMOFs are mainly reflected in fluorescent changes. As the functional groups of MIL-53(Al)-2.0G PAMAM could provide abundant coordination sites for heavy metal ions, the distance between the heavy metal ions and PAMAM-grafted MOFs greatly decreased, which significantly encouraged electron transfer and quenched the fluorescence.

**Figure 7:**
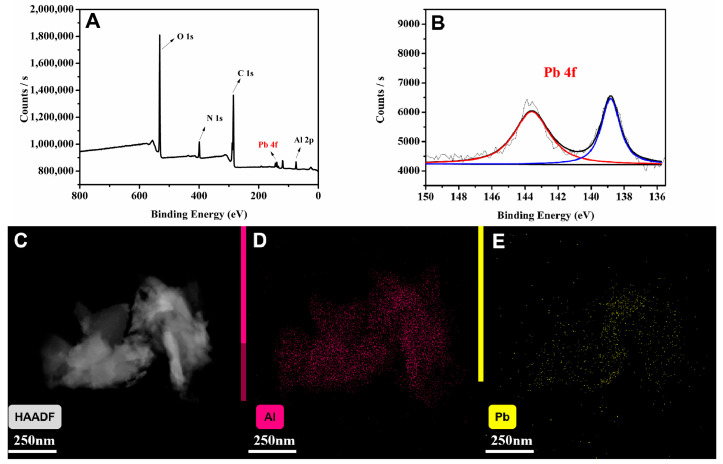
(**A**) Full XPS spectrum of Pb^2+^ quenched MIL-53(Al)-2.0G PAMAM; (**B**) Pb 4f spectra after Pb^2+^ treatment (The red and blue line is the orbital peak fitting result of Pb 4f, and two black lines are the baseline and the total result of all peak fits, respectively); (**C**–**E**) EDS-mapping of Pb^2+^ quenched MIL-53(Al)-2.0G PAMAM.

In order to verify the assumed mechanism, MIL-53(Al)-2.0G PAMAM materials were collected and characterized by XPS after being quenched by Pb^2+^. The states of the contained elements are shown in Figure 7. Peaks in 531.41, 399.20, 284.80 and 74.03 eV corresponded to O 1s, N 1s, C 1s and Al 2p in MIL-53(Al)-2.0G PAMAM, respectively (Figure 7A). After being quenched by Pb^2+^, the peaks of Pb appeared distinctly (Figure 7B). At the same time, EDS-mapping analysis was performed on the quenched MIL-53(Al)-2.0G PAMAM materials. According to the result, the quenched fluorescent materials contained Al and Pb elements (Figure 7C–E). Al exists in the material as the skeleton structure, while Pb is the metal ion adsorbed during fluorescence quenching, which verifies the coordination of metal ions and the electron transfer mechanism.

### 3.5. Fluorescence Enhancement Property for Other MIL MOFs

The results of the current study showed that the fluorescence intensity of MIL-53(Al)-NH_2_ could be further enhanced by PAMAM grafting, providing higher sensitivity in detecting heavy metal ions. To explore the general applicability of this approach, other water-stable MIL MOFs MIL-53(Fe)-NH_2_) were synthesized and subsequently grafted with 2.0G PAMAM dendrimers. Fluorescence intensities of the obtained materials were investigated and compared with that of raw MOFs. According to the results in Figure S3, PAMAM grafting could also increase the fluorescence intensity of MIL-53(Fe)-NH_2_. Therefore, it could be assumed that the proposed method in this study is universal for analogous water-stable MIL LMOFs.

## 4. Conclusions

In this work, water-stable MOFs named MIL-53(Al)-NH_2_ were synthesized and functionalized to have high fluorescence intensities via morphology control and PAMAM grafting. It was found that MIL-53(Al)-NH_2_ materials synthesized with different solvents had different morphologies, sizes and fluorescence properties. Due to ICT mechanism, PAMAM dendrimers further improved the fluorescence intensity of MIL-53(Al)-NH_2_. With the enhanced fluorescence intensity, the proposed materials showed good linearity (R^2^ = 0.9925–0.9990) and satisfactory sensitivity (LOD = 1.1–8.6 μmol) in heavy metal ions detection. Moreover, such a fluorescence enhancement approach was proved to be universal for analogous MIL MOFs, which is of great significance for the preparation of highly sensitive fluorescent MOFs probes in the future.

## Figures and Tables

**Table 1:** Analytical performance of the PAMAM-grafted MOFs and comparison with the reported raw MIL MOFs sensors.

Heavy Metal Ions	MIL-53-2.0G PAMAM			Published Raw MIL MOFs (Ref. [22])		
	Linear Range (μM)	R^2^	LOD (μM)	Linear Range (mM)	R^2^	LOD (μM)
Pb^2+^	5–50	0.9947	1.1	0.01–1	0.9993	5.2
Cu^2+^	5–100	0.9942	1.1	0.01–10	0.9933	1.6
Fe^3+^	10–100	0.9990	2.9	0.01–0.2	0.9934	1.8
Co^2+^	20–80	0.9925	8.6	*	*	*

* Co^2+^ was not detected.

**Table 2:** Recovery rates of the spiked Pb^2+^ and Fe^3+^ in tap water, drinking water and tea samples.

Spiked Ions	Sample	Spiked (mM)	Determined (mM)	Recovery
Pb^2+^	Tap water	0.05	0.0441 ± 0.0145	88.2%
	Drinking water	0.05	0.0470 ± 0.0014	94.0%
	Tea	0.05	0.0327	65.4%
Fe^3+^	Tap water	0.05	0.0477 ± 0.0002	95.4%
	Drinking water	0.05	0.0475 ± 0.0007	95.0%
	Tea	0.05	0.0166	33.2%

## Data Availability

Data available on request from the authors.

## Supplementary Materials

The following supporting information can be downloaded at: https://www.mdpi.com/article/10.3390/polym15163444/s1, Figure S1: Extent of the fluorescence response of MIL-53-2.0G PAMAM to various metal ions; Figure S2: Fluorescence response spectra of heavy metal ions with different concentrations; Figure S3: Fluorescence emission spectra of MIL-53(Fe)-NH_2_ and MIL-53(Fe)-2.0G PAMAM; Table S1: Element analysis for 1.0G PAMAM and 2.0G PAMAM.

## References

[1] Yang Z., Liu H.W., Li J., Yang K., Zhang Z.Z., Chen F.J., Wang B.D. (2020). High-throughput metal trap: Sulfhydryl-functionalized wood membrane stacks for rapid and highly efficient heavy metal ion removal. Acs. Appl. Mater. Inter..

[2] Ebrahimi-Najafabadi H., Pasdaran A., Bezenjani R.R., Bozorgzadeh E. (2019). Determination of toxic heavy metals in rice samples using ultrasound assisted emulsification microextraction combined with inductively coupled plasma optical emission spectroscopy. Food Chem..

[3] Li A.Y., Deng H., Jiang Y.H., Ye C.H., Yu B.G., Zhou X.L., Ma A.Y. (2020). Superefficient removal of heavy metals from wastewater by Mg-loaded biochars: Adsorption characteristics and removal mechanisms. Langmuir.

[4] Manousi N., Deliyanni E., Zachariadis G. (2020). Multi-element determination of toxic and nutrient elements by ICP-AES after dispersive solid-phase extraction with modified graphene oxide. Appl. Sci..

[5] Xing G.W., Sardar M.R., Lin B.X., Lin J.M. (2019). Analysis of trace metals in water samples using NOBIAS chelate resins by HPLC and ICP-MS. Talanta.

[6] Du X.X., Liu Y.J., Wang F., Zhao D.Y., Gleeson H.F., Luo D. (2021). A fluorescence sensor for Pb^2+^ detection based on liquid crystals and aggregation-induced emission luminogens. Acs. Appl. Mater. Inter..

[7] Li C.H., Zhu L., Yang W.X., He X., Zhao H.P., Tang W.Z., Yue T.L., Li Z.H. (2020). Post-functionalized Al-based metal-organic frameworks for fluorescent detection of total iron in food matrix. J. Food Compos. Anal..

[8] Yin H., Truskewycz A., Cole I.S. (2020). Quantum dot (QD)-based probes for multiplexed determination of heavy metal ions. Microchim. Acta.

[9] Zare H., Ghalkhani M., Akhavan O., Taghavinia N., Marandi M. (2017). Highly sensitive selective sensing of nickel ions using repeatable fluorescence quenching-emerging of the CdTe quantum dots. Mater. Res. Bull..

[10] Wang K.C., Li Y.P., Xie L.H., Li X.Y., Li J.R. (2022). Construction and application of base-stable MOFs: A critical review. Chem. Soc. Rev..

[11] Xie L.H., Xu M.M., Liu X.M., Zhao M.J., Li J.R. (2020). Hydrophobic metal-organic frameworks: Assessment, construction, and diverse applications. Adv. Sci..

[12] Saraci F., Quezada-Novoa V., Donnarumma P.R., Howarth A.J. (2020). Rare-earth metal-organic frameworks: From structure to applications. Chem. Soc. Rev..

[13] Ren K., Guo X.F., Tang Y.J., Huang B.H., Wang H. (2020). Size-controlled synthesis of metal-organic frameworks and their performance as fluorescence sensors. Analyst.

[14] He J., Xu F., Tian Y., Li C., Hou X. (2020). Atmospheric low-temperature plasma for direct post-synthetic modification of UiO. Chem. Commun..

[15] Jindal S., Moorthy J.N. (2022). Zwitterionic luminescent 2D metal-organic framework nanosheets (LMONs): Selective turn-on fluorescence sensing of dihydrogen phosphate. Inorg. Chem..

[16] Wang Q.H., Qi X.D., Chen H.Y., Li J.G., Yang M., Liu J., Sun K., Li Z.H., Deng G.W. (2022). Fluorescence determination of chloramphenicol in milk powder using carbon dot decorated silver metal-organic frameworks. Microchim. Acta.

[17] Wang X.J., Mal A., Gui B., Wang C. (2023). Tuning energy transfer in metal-organic frameworks for fluorescence turn-on sensing of Hg(II) ions. Chin. J. Chem..

[18] Saedi Z., Roushani M., Khaleghian-Moghadam R., Darabi A. (2022). Selective and sensitive Detection of Cu^2+^ in aqueous solution based on cation exchange by Metal-Organic framework TMU-16 as a fluorescent sensor. J. Lumin..

[19] Chen L., Wang C.L., Zhu C.Y., Li P., Gao W., Li J.Y., Zhang X.M. (2022). Recyclable luminescence sensor for Cu^2+^, Cr_2_O_7_^2−^ and CrO_4_^2−^ in water and acid/base vapor response based on water-stable bipyridyl-based Ln-MOFs. J. Solid State Chem..

[20] Mohan B., Tao Z.Y., Kumar S., Xing T.T., Ma S.X., Huang W.B., Yang X.P., You H.Z., Ren P. (2022). Water- and pH-stable methylthio-containing metal-organic frameworks as luminescentsensors for metal-ion detection. Cryst. Growth Des..

[21] Liu B.T., Vikrant V., Kim K.H., Kumar V., Kailasa S.K. (2020). Critical role of water stability in metal-organic frameworks and advanced modification strategies for the extension of their applicability. Environ. Sci. Nano.

[22] Lv S.W., Liu J.M., Li C.Y., Zhao N., Wang Z.H., Wang S. (2019). A novel and universal metal-organic frameworks sensing platform for selective detection and efficient removal of heavy metal ions. Chem. Eng. J..

[23] Yasir A.T., Benamor A., Hawari A.H., Mahmoudi E. (2023). Poly (amido amine) dendrimer based membranes for wastewater treatment-A critical review. Chem. Eng. Sci..

[24] Pandey M., Lang H.X., Loh J.S., Chai Y.L., Tee H.L., Mayuren J., Candasamy M., Gorain B., Jain N., Gupta G. (2023). Dendrimer platform against prostate cancer: Recent update on new horizon of treatment. J. Drug Deliv. Sci. Tec..

[25] Guo D.D., Zhou X.Q., Muhammad N., Huang S.H., Zhu Y. (2022). An overview of poly (amide-amine) dendrimers functionalized chromatographic separation materials. J. Chromatogr. A.

[26] Ji Z.C., Sun H.Y., Zhu Y.F., Zhang D.D., Wang L.H., Dai F.Y., Zhao Y.P., Chen L. (2021). Enhanced selective removal of lead ions using a functionalized PAMAM@UiO-66-NH_2_ nanocomposite: Experiment and mechanism. Micropor. Mesopor. Mat..

[27] Georgiev N.I., Asiri A.M., Qusti A.H., Alamry K.A., Bojinov V.B. (2014). A pH sensitive and, selective ratiometric PAMAM wavelength-shifting bichromophoric system based on PET, FRET and ICT. Dyes Pigments.

[28] Guo D.D., Huang S.H., Zhu Y. (2022). The adsorption of heavy metal ions by Poly (Amidoamine) dendrimer-functionalized nanomaterials: A review. Nanomaterials.

[29] Tomalia D.A., Naylor A.M., Goddard W.A. (1990). Starburst dendrimers-molecular-level control of size, shape, surface-chemistry, topology, and flexibility from atoms to macroscopic matter. Angew. Chem. Int. Edit..

[30] Wei J.Y., Zhang D., Zhang L.X., Ouyang H., Fu Z.F. (2019). Alkaline hydrolysis behavior of metal-organic frameworks NH_2_-MIL-53(Al) employed for sensitive immunoassay via releasing fluorescent molecules. ACS Appl. Mater. Inter..

[31] Xiong W.P., Zeng Z.T., Li X., Zeng G.M., Xiao R., Yang Z.H., Zhou Y.Y., Zhang C., Cheng M., Hu L. (2018). Multi-walled carbon nanotube/amino-functionalized MIL-53(Fe) composites: Remarkable adsorptive removal of antibiotics from aqueous solutions. Chemosphere.

[32] Yusuf V.F., Malek N.I., Kailasa S.K. (2022). Review on metal-organic framework classification, synthetic approaches, and Iinfluencing factors: Applications in energy, drug delivery, and wastewater treatment. ACS Omega.

[33] Lu T., Zhang L.C., Sun M.X., Deng D.Y., Su Y.Y., Lv Y. (2016). Amino-functionalized metal-organic frameworks nanoplates-based energy transfer probe for highly selective fluorescence detection of free chlorine. Anal. Chem..

[34] Cui B.F., Zhang L., Zhou H.D., Zhang J.Y., Chen J.M. (2011). Preparation and tribological properties of self-assembled poly(amide amine)-Cu nanofilm on silicon. Surf. Interface Anal..

[35] Riascos H., Zambrano G., Prieto P., Arroyave M., Devia A., Galindo H. (2004). Correlation between plasma characterization and growth of fullerene-like CNx thin films deposited by pulsed laser ablation. Surf. Coat. Tech..

[36] Hu Y., Goodeal N., Chen Y., Ganose A.M., Palgrave R.G., Bronstein H., Blunt M.O. (2016). Probing the chemical structure of monolayer covalent-organic frameworks grown via Schiff-base condensation reactions. Chem. Commun..

[37] Dong C.L., Li M.F., Yang T., Feng L., Ai Y.W., Ning Z.L., Liu M.J., Lai X., Gao D.J. (2021). Controllable synthesis of Tb-based metal-organic frameworks as an efficient fluorescent sensor for Cu^2+^ detection. Rare Met..

[38] Pu L. (2012). Enantioselective fluorescent sensors: A tale of BINOL. Acc. Chem. Res..

[39] Devadoss C., Bharathi P., Moore J.S. (1996). Energy transfer in dendritic macromolecules: Molecular size effects and the role of an energy gradient. J. Am. Chem. Soc..

[40] Ren M.J., Wang H., Liu Y.Y., Ma Q., Jia W.J., Liu M.Z., Wang H.J., Lu Y.C. (2020). Fluorescent determination of mercury (II) and glutathione using amino-MIL-53(Al) nanosheets. Anal. Lett..

[41] Bae W., Yoon T.Y., Jeong C. (2021). Direct evaluation of self-quenching behavior of fluorophores at high concentrations using an evanescent field. PLoS ONE.

[42] Li Y.X., Tang L., Zhu C.X., Liu X.Y., Wang X., Liu Y. (2022). Fluorescent and colorimetric assay for determination of Cu(II) and Hg(II) using AuNPs reduced and wrapped by carbon dots. Microchim. Acta.

