# Supplementary material for: Polyamidoamine Dendrimers Functionalized Water-Stable Metal–Organic Frameworks for Sensitive Fluorescent Detection of Heavy Metal Ions in Aqueous Solution
Source: Polymers (Basel). 2023 Aug 18;15(16):3444. doi: 10.3390/polym15163444 (PMC10458630; doi:10.3390/polym15163444)
Supplement: Supplementary file 1 [file polymers-15-03444-s001.zip › polymers-2542244-supplementary.pdf]

Polyamidoamine dendrimers functionalized water stable metal organic frameworks for sensitive fluorescent detection of heavy metal ions in aqueous solution

Dandan Guo [1,*], Nadeem Muhammad [2], Shuxin Yu[1], Jinhui Wang[1], Shaohua Huang[1,3*] and Yan Zhu [4,*]

[1] Institute of Drug Discovery and Technology, Ningbo University, Ningbo 315211, China
[2] Department of Environmental Engineering, Wuchang University of Technology, Wuhan 430223, China
[3] Qian Xuesen Collaborative Research Center of Astrochemistry and Space Life Sciences, Ningbo University, Ningbo 315211, China
[4] Department of Chemistry, Xixi Campus, Zhejiang University, Hangzhou 310028, China
*Correspondence: guodandan@nbu.edu.cn (Dandan Guo); huangshaohua@nbu.edu.cn (Shaohua Huang); zhuyan@zju.edu.cn (Yan Zhu)

1. Synthesis of MIL-53(Fe)-NH_2

MOFs MIL-53(Fe)-NH_2 was prepared by the hydrothermal method[1]: 0.36 g $FeCl_3 \cdot 6H_2O$ and 0.25 g NH_2-BDC were dissolved in 30 mL DMF. The mixture was placed in a 50 mL Teflon-lined autoclave and maintained at 170 °C for 24 hours. The resulting products were separated from reaction mixture by centrifugation and washed by DMF and methanol successively. Finally, the obtained products were dried at 70 °C in vacuum overnight.

2. Figures

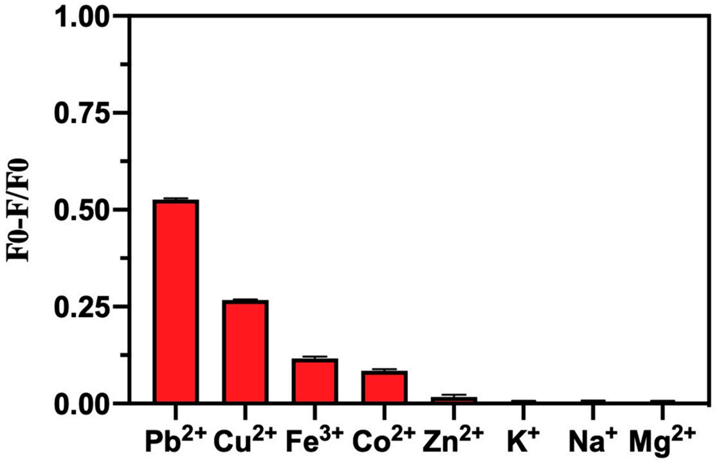

Figure S1 Extent of the fluorescence response of MIL-53-2.0G PAMAM to various metal ions (excitation slit: 2.5 nm; emission slit: 2.5 nm)

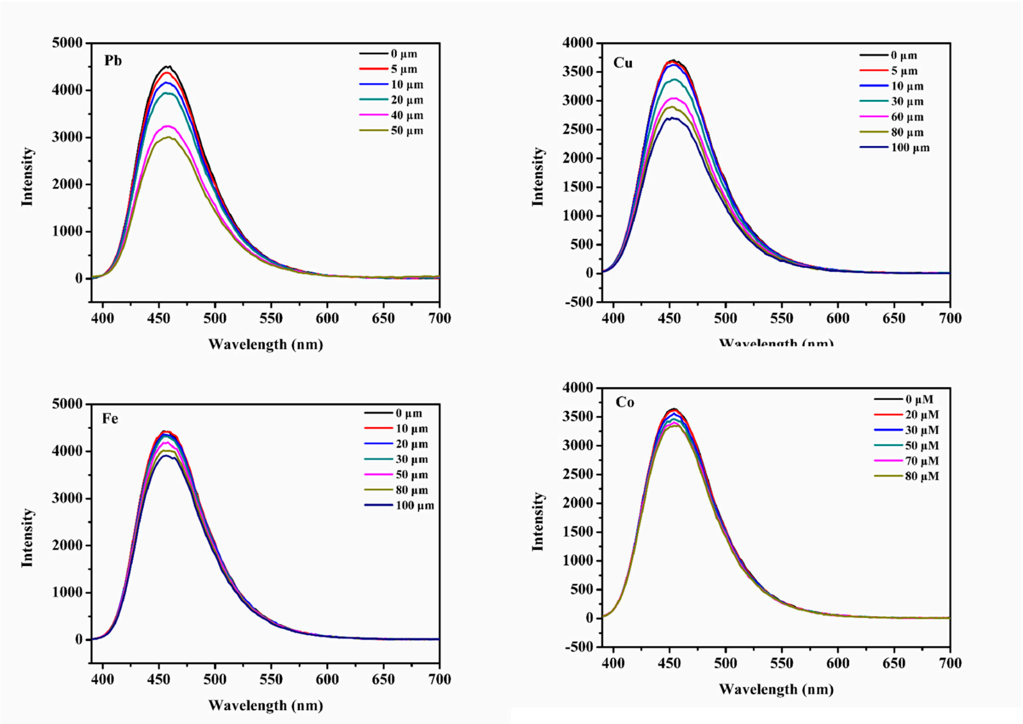

Figure S2 Fluorescence response spectra of heavy metal ions with different concentrations

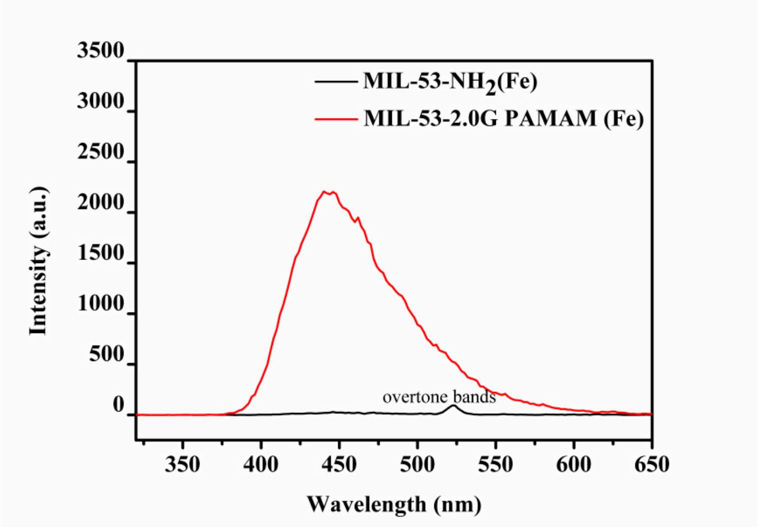

Figure S3 Fluorescence emission spectra of MIL-53(Fe)-NH_2 and MIL-53(Fe)-2.0G PAMAM (concentration: 1 mg/mL, excitation wavelength: 260 nm, excitation slit: 2.5 nm; emission slit: 5 nm);

3. Tables

Table. S1 Elemnet analysis for 1.0G PAMAM and 2.0G PAMAM

	C%	H%	N%
1.0G PAMAM	42.51	10.56	30.51
2.0G PAMAM	45.32	9.74	28.49
